# Retinal Neurovascular Coupling: From Mechanisms to a Diagnostic Window into Brain Disorders

**DOI:** 10.3390/cells14221798

**Published:** 2025-11-16

**Authors:** Wen Shen

**Affiliations:** Department of Biomedical Science, Charles E Schmidt College of Medicine, Florida Atlantic University, Boca Raton, FL 33431, USA; wshen@health.fau.edu

**Keywords:** neurovascular unit, retinal neurovascular coupling, Alzheimer’s disease, Parkinson’s disease, Huntington’s disease, stroke

## Abstract

Retinal neurovascular coupling reflects the precise coordination between neuronal activity, glial support, and vascular responses, mirroring key neurovascular mechanisms in the brain. This review emphasizes the cellular and molecular processes underlying retinal neurovascular coupling and positions the retina as a sensitive and accessible model for investigating neurovascular function in the brain. It highlights how parallel neurovascular degeneration in the brain and retina provides critical insights into the pathophysiology of neurodegenerative and vascular disorders. Advances in retinal imaging, including functional optical coherence tomography (fOCT), OCT angiography (OCTA), and functional electrophysiology, offer unprecedented opportunities to detect early neuronal and vascular dysfunction, establishing the retina as a non-invasive biomarker for early detection, disease monitoring, and therapeutic evaluation in Alzheimer’s, Parkinson’s and Huntington’s disease, and stroke. By integrating structural, functional, and mechanistic approaches, the review emphasizes the retina’s potential as a translational platform bridging basic science and clinical applications in neurovascular research.

## 1. Introduction

**Figure 1:**
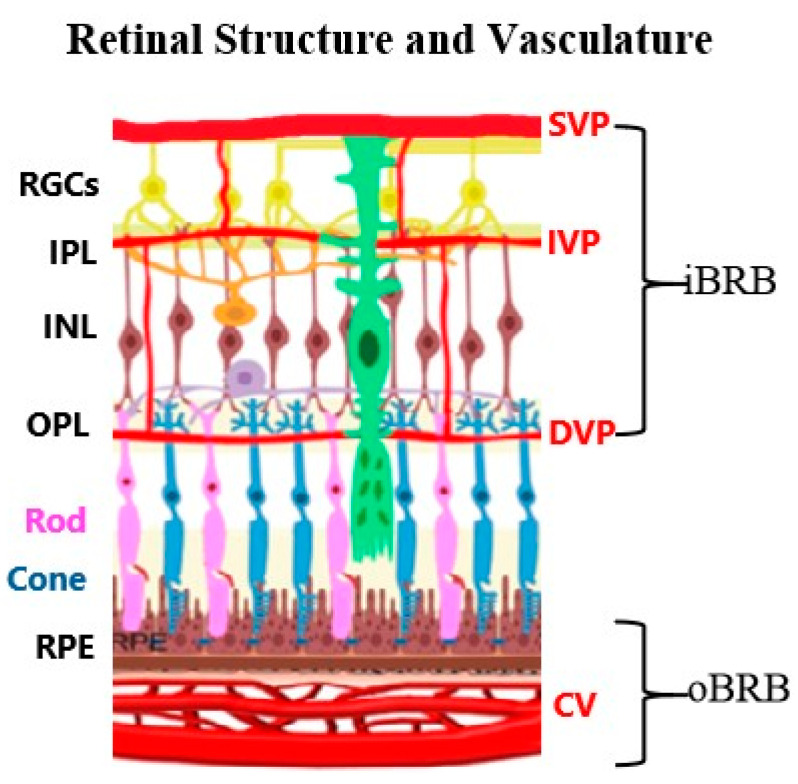
Retinal layers and vasculature organization. The inner retinal circulation consists of the superficial (SVP), intermediate (IVP), and deep vascular plexuses (DVP), while the outer retina, including photoreceptors and retinal pigment epithelium (RPE), is supplied by the choroidal vasculature (CV). RGCs, retinal ganglion cells; IPL, inner plexiform layer; INL, inner nuclear layer; OPL, outer plexiform layer.

The retina shares core neurovascular features with the brain, yet remains optically accessible, which offers a unique advantage for studying neurovascular coupling in real time. Neurovascular coupling, the mechanism that matches neural activity with proportional blood flow, is orchestrated by the neurovascular unit, a coordinated ensemble of neurons, glia, pericytes, and endothelial cells [1]. In the retina, this unit not only mediates activity-dependent vascular responses but also governs endothelial tight junctions that compose the inner blood–retina barrier (iBRB). Similar to the brain’s blood–brain barrier (BBB), the iBRB tightly regulates vascular permeability to maintain neural homeostasis. The retinal vasculature is organized into two distinct barrier systems: the outer blood–retina barrier (oBRB), formed by the retinal pigment epithelium and choroidal vasculature, and the iBRB, maintained by the retinal neurovascular unit (Figure 1) [2,3]. Serving as both a specialized sensory organ and an accessible model system, the retina integrates photoreceptor activity with downstream neural processing to generate visual signals, relying on tightly regulated neurovascular coupling to meet metabolic demands.

While the retina and brain share many structural and cellular features, their vascular architectures vary to meet distinct metabolic and signaling demands. The inner retinal vasculature exhibits a laminar vascular organization composed of three distinct layers: the superficial-, intermediate-, and deep-vascular plexus, each with unique structural and functional roles, whereas the brain’s vasculature forms a complex, interconnected network of capillaries branching at various angles to support diverse neuronal circuits [4,5]. Despite these architectural differences, the inner retinal neurovascular interactions and the iBRB preserve the fundamental molecular and cellular signaling pathways and vascular organization of the brain [6,7]. Moreover, the three-plexus vascular network of the retina provides an ideal platform for investigating neurovascular coupling in both health and disease. In recent years, increasing evidence has shown that neurovascular coupling dysfunction is a unifying feature in a range of central nervous system (CNS) disorders, from Alzheimer’s, Parkinson’s, Huntington’s disease, to stroke, and that similar pathological changes are detectable in the retina [1,8,9,10,11]. Accordingly, multiple advanced imaging techniques and computational analytical approaches have been employed for retinal assessment, including dynamic vessel analysis (DVA), adaptive optics ophthalmoscopy, functional optical coherence tomography (fOCT), OCT angiography (OCTA), and integrated electrophysiology [12,13,14,15]. As a result, these technological advances have deepened our understanding of neurovascular coupling mechanisms in the retina, thereby providing insights directly translatable to our understanding of neurovascular coupling and its dysregulation in health and diseased brains.

Taken together, this review emphasizes fundamental signaling pathways of neurovascular coupling, recent discoveries, methodological frontiers, disease-relevant insights, and translational opportunities. By positioning the retina as both a sensory organ and a model system, it offers a unique perspective on how neuronal activity, blood flow, and glial/astrocytes are integrated to regulate brain function.

## 2. Retinal Neurovascular Unit: Mechanism and Diversity

### 2.1. Cellular Components of Neurovascular Unit

**Figure 2:**
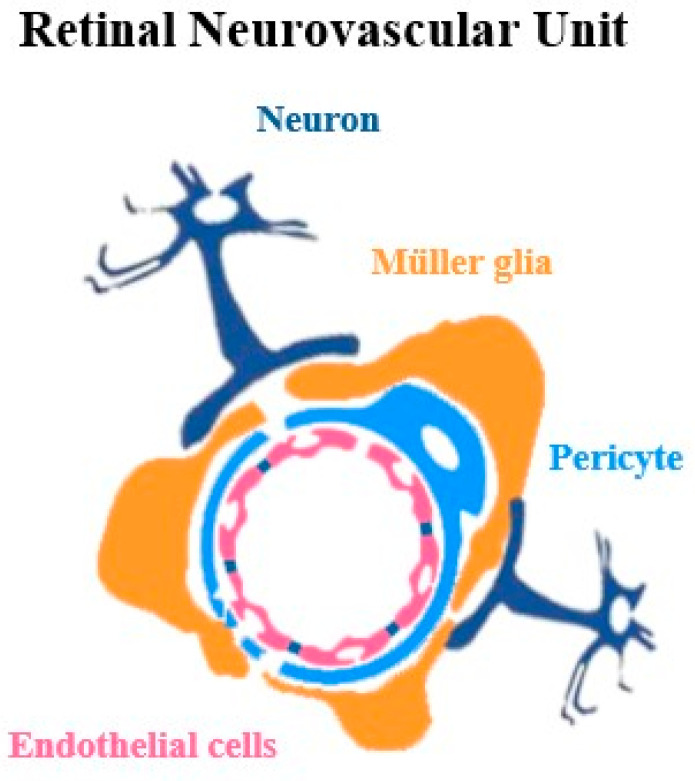
Composition of the retinal neurovascular unit in the inner retinal circuitry.

The retinal neurovascular unit is a highly integrated system composed of neurons, glial cells (including astrocytes, Müller cells, and microglia), and vascular elements (endothelial cells, pericytes, and vascular smooth muscle cells), which operate synergistically to preserve retinal homeostasis and sustain optimal visual function (Figure 2). In addition, the extracellular matrix (ECM) supports and regulates interactions between cells and blood vessels, helping the unit function normally. Retinal neurovascular coupling occurs within this unit: photoreceptors, bipolar cells, and ganglion cells initiate signaling by releasing glutamate, activating Müller glia and astrocytes, and interneurons. These, in turn, release vascular mediators including adenosine triphosphate (ATP) and nitric oxide (NO) that regulate vascular tone [16,17]. Glia and astrocytes in the unit act as pivotal intermediaries, sensing neural activity and releasing vasoactive factors that adjust vessel diameter to ensure blood flow meets the metabolic demands of active neurons [18,19,20,21]. Beyond this, glial cells, in particular, provide metabolic support, buffer extracellular potassium and other ions, and remove excess neurotransmitters, thereby maintaining homeostasis within the retinal microenvironment [18,22]. Simultaneously, the vascular elements of the unit, endothelial cells, pericytes, and vascular smooth muscle cells, along with the basement membrane, respond to these neuronal and glial signals in a coordinated manner [23]. Endothelial cells adjust iBRB permeability and mediate transcellular and paracellular transport, while pericytes regulate capillary diameter and stabilize vessel structure. Vascular smooth muscle cells modulate arteriolar tone to control blood flow [24]. Overall, this tightly coordinated system ensures that retinal blood flow dynamically adapts to neuronal activity, delivering adequate oxygen and nutrients while preventing harmful fluctuations in the retinal microenvironment [2,19].

### 2.2. Heterogeneity of Neurovascular Unit

Extensive research indicates that the mechanisms underlying retinal neurovascular coupling closely parallel those in the brain, reflecting the conserved organization of the neurovascular unit across the CNS [25]. Yet, despite this shared framework, the neurovascular unit exhibits considerable regional heterogeneity driven by differences in cellular composition, structural organization, and metabolic demands. Within the eye, retinal capillaries form superficial, intermediate, and deep plexuses that supply the inner retinal neurons, while the choroid contains a fenestrated, high-flow vascular network that nourishes photoreceptors. The iris–ciliary body vasculature, in turn, supports aqueous humor production. These vascular compartments differ in endothelial features, pericyte coverage, basement membrane composition, and barrier properties, highlighting the specialization of the ocular neurovascular unit and its variable susceptibility to disease [16,26]. Importantly, the distinctions extend beyond ocular tissues to the broader CNS. In the retina, Müller cells, radial glia spanning the retinal layers, coordinate neuronal support, metabolism, and blood flow, whereas in the brain, astrocytes fulfill similar roles through perivascular end-feet that regulate BBB, vascular tone, and metabolic exchange, thereby ensuring adequate oxygen and nutrient delivery [19,27].

Additional distinctions in the neurovascular unit include variations in astrocytic end-foot density and morphology, pericyte density, endothelial tight junction complexity, and basement membrane composition across cortical gray matter, white matter, brainstem nuclei, and retinal layers [27]. These structural differences translate into regional variation in neurovascular coupling efficiency, barrier permeability, and susceptibility to inflammatory or ischemic injury. Such heterogeneity not only allows local adaptation to specific energy demands and signaling environments, but it also contributes to selective vulnerability in disease, for example, the selective retinal layer injury in diabetic retinopathy or preferential involvement of white matter in cerebral small-vessel disease [27,28,29]. Recent single-cell transcriptomic studies further highlight the diversity of endothelial cells, revealing distinct molecular signatures for arteriolar, capillary, and venular segments, along with differential responses to pericyte loss [30,31]. These include segment-specific pericyte depletion, altered tight junction protein expression, and shifts in inflammatory and metabolic gene profiles, underlining the variable vulnerability and adaptive capacity of neurovascular components across the vascular tree [26,27]. Recognizing this heterogeneity is therefore essential for developing targeted therapies that address region-specific neurovascular pathophysiology.

## 3. Signal Pathways of Neurovascular Coupling

The regulation of retinal blood flow relies on tightly orchestrated signaling pathways that link neuronal activity to vascular responses in neurovascular coupling. Multiple mechanisms, including NO production, purinergic signaling, ion/metabolic pathways, and vascular endothelial growth factor (VEGF), act in concert within the retinal neurovascular unit to translate synaptic activity into dynamic changes in vascular tone. Together, these pathways ensure precise matching of blood supply to the metabolic demands of visual processing and safeguard retinal homeostasis.

### 3.1. NO Signaling Pathways

During visual activity, glutamatergic input activates N-methyl-D-aspartate (NMDA) receptors in bipolar, amacrine, and ganglion cells. These neurons, which express the neuronal isoform of nitric oxide synthase (nNOS), produce NO upon depolarization [32,33,34]. Once generated, NO readily diffuses to the smooth muscle cells of adjacent arterioles, where it modulates vascular tone [32,33]. Because of this direct action, NO production is regarded as a central mechanism in neurovascular coupling [35]. Furthermore, Müller glia expressing inducible nitric oxide synthase (iNOS) can generate NO under conditions of intensified neuronal activity or metabolic stress, thereby providing further support for vascular regulation [36,37]. These neuronal and glial cues guide endothelial cells and vascular smooth muscle cells to execute precise vascular adjustments during neurovascular coupling, with NO acting as a key mediator of vasodilation. NO is synthesized not only by neurons and glia but also by endothelial cells through the activation of endothelial nitric oxide synthase (eNOS) [38]. Upon release, NO diffuses into adjacent vascular smooth muscle cells, where it activates soluble guanylate cyclase (sGC), increasing cyclic GMP (cGMP) levels [39]. Elevated cGMP activates protein kinase G (PKG), which phosphorylates multiple targets, including myosin light chain phosphatase and calcium-handling proteins, leading to reduced intracellular calcium and dephosphorylation of myosin light chains. These changes relax smooth muscle fibers, dilate vessels, and increase local blood flow to meet metabolic demand [32,40].

In both the brain and retina, neuronal nNOS and glial iNOS generate NO in response to activity, while endothelial eNOS contributes additional NO. This NO diffuses to nearby vascular smooth muscle cells, activating sGC → cGMP → PKG signaling to relax vessels and increase blood flow. In the retina, this pathway is further stratified across the superficial, intermediate, and deep plexuses, supporting different neuronal layers and metabolic demands. For example, the deep capillary plexus supplies the inner segment/outer segment region of photoreceptors, while the superficial plexus serves the ganglion cell layer. This stratified microvascular architecture allows finely tuned, layer-specific blood flow regulation: NO-driven vasodilation may predominate in the superficial plexus during ganglion cell activity [17], while other mediators may complement NO signaling in deeper layers to support photoreceptor metabolism.

### 3.2. Signaling Pathways for ATP in Vasodilation and Constriction

Alongside this NO-dependent pathway, purinergic signaling provides another important layer of control over neurovascular responses. Müller cells release ATP upon activation of metabotropic glutamate receptors, and ATP itself can act as a feed-forward activator in the retina [20]. During flickering light stimulation, amacrine and ganglion cells also release ATP, which activates P_2X_ and P_2Y_ receptors on the endfeet of Müller glia and astrocytes that wrap around retinal blood vessels. Activation of P_2X_ receptors promotes calcium influx, whereas stimulation of P_2Y_ receptors engages the phospholipase C (PLC)—inositol triphosphate (IP_3_) pathway, leading to calcium release from intracellular stores [21,41,42]. These calcium signals trigger the release of vasoactive metabolites, including arachidonic acid derivatives, such as prostaglandin E_2_ (PGE_2_) and epoxyeicosatrienoic acids (EETs), which increase blood flow. PGE_2_ acts by binding to specific G-protein-coupled E-prostanoid (EP) receptors on endothelial cells and smooth muscle cells, activating adenylyl cyclase (AC), raising intracellular cyclic adenosine monophosphate (cAMP) levels, and activating protein kinase A (PKA), ultimately promoting vasodilation [18,43]. EETs, in turn, increase the activity of calcium-activated potassium (BK_Ca_) channels in vascular smooth muscle cells and endothelial cells, causing hyperpolarization, relaxation, and vasodilation, although their precise receptor remains incompletely defined [21,42,44]. In contrast to these vasodilatory mechanisms, vasoconstriction is mediated in part by Müller cells and astrocytes releasing 20-HETE, an arachidonic acid derivative [45]. A key aspect of its action involves the inhibition of BK_Ca_ channels in vascular smooth muscle cells through signaling pathways that include activation of protein kinase C (PKC), mitogen-activated protein kinase (MAPK), and Src-type tyrosine kinases (c-Src) [46]. By blocking potassium efflux required for smooth muscle relaxation, 20-HETE promotes vasoconstriction, providing a complementary mechanism that balances retinal blood flow alongside NO- and ATP-mediated vasodilation. In addition to its activity-dependent regulation, ATP also contributes to baseline vascular tone: tonic release from astrocytes can induce mild vasoconstriction under resting conditions, which is subsequently overridden by vasodilation during neuronal activity [16]. Finally, the breakdown of ATP to adenosine further refines vascular tone through activation of A_1_ and A_2_ adenosine receptors [47,48,49]. Adenosine receptors (A_2A_, A_2B_) further enhance vasodilation through cAMP-dependent pathways [50,51]. These are well-characterized vasodilation mechanisms playing central roles in controlling vascular tone. In the retina, the stratified microvascular organization enables finely tuned regulation of blood flow: ATP/adenosine signaling appears to be particularly important in the deep capillary plexus, where high synaptic activity triggers ATP release from neurons and Müller glia, which in turn modulates pericyte and endothelial function to adjust capillary diameter [52]. In contrast, NO signaling predominates in the superficial plexus, where ganglion cell activity and astrocyte-neuron interactions drive rapid vasodilation. This division of labor enables the retina to match blood supply to local metabolic demand across its distinct vascular layers.

### 3.3. Ions in Regulation of Blood Flow

In addition to these multifaceted regulators, ion and metabolic coupling mechanisms are also critical for fine-tuning blood flow and ensuring adequate energy supply during neuronal activity. In both the brain and retina, K^+^ efflux from active neurons and glia alters extracellular potassium levels, thereby modulating vascular tone [53,54]. The inward rectifying K^+^ channel, Kir4.1 channels, abundantly expressed in Müller cells of the retina and astrocytes in the brain, play a central role in buffering extracellular potassium and relaying activity-dependent signals to vascular smooth muscle cells and pericytes [48,53]. Complementing this, bicarbonate transporters (e.g., Na^+^/HCO_3_^−^ cotransporters) and carbonic anhydrases regulate extracellular pH and CO_2_, further influencing vasodilation and constriction [55,56]. In parallel, local elevations in extracellular K^+^ can trigger vasodilation via inward rectifier K^+^ channels in vascular cells, while Ca^2+^ signals within neurons and Müller glia coordinate the release of vasoactive mediators such as NO, ATP, and prostaglandins. Together, these interconnected signaling mechanisms integrate neuronal, glial, and astrocytic activity to precisely regulate neurovascular responses.

### 3.4. VEGF Signaling Pathways

Another key regulator is VEGF, which promotes endothelial survival, barrier integrity, and angiogenic potential. VEGF also enhances NO production by activating eNOS through VEGF receptor-2 (VEGFR2) signaling, with the resulting NO release contributing to vasodilation and improved perfusion [57]. VEGF also stimulates vascular permeability by reorganizing tight and adherens junctions, allowing dynamic exchange between the circulation and surrounding tissue [58]. In addition, VEGF drives retinal angiogenesis by binding to its receptors on endothelial cells, stimulating their sprouting, migration, and new capillary formation, thereby adapting blood supply to local metabolic demands to improve neovascularization [59]. However, when overactivated, as in diabetic retinopathy, ischemia, or inflammation, excessive VEGF disrupts tight junctions of the iBRB and promotes abnormal neovascularization [60]. Studies in the brain further show that following injury, such as stroke or brain tumors, increased VEGF expression enhances angiogenesis and promotes BBB, which is thought to represent an attempt to repair damaged tissue by enhancing blood supply and nutrient delivery to the affected area [61]. Thus, VEGF functions as both a vital physiological mediator of blood flow and a pathological driver of vascular dysregulation and dysfunction.

### 3.5. Signal Pathways of Pericytes

Within the neurovascular unit, pericytes work with endothelial cells to fine-tune capillary blood flow through contractile regulation. These specialized mural cells, embedded within the basement membrane and in close contact with endothelial cells, sense neuronal and glial signals through diverse receptor pathways, including purinergic (P_2X_/P_2Y_), adrenergic (A_1_, A_2_), and endothelin receptor B (ETR_B_) [62]. Pericytes respond to vasodilatory mediators such as NO, PGE_2_, and EETs with relaxation, thereby promoting capillary dilation, whereas vasoconstrictors, such as thromboxane A_2_ or endothelin-1, acting through ETR_A_, induce vasoconstriction [63,64]. They also contribute to extracellular K^+^ sensing, where elevated extracellular K^+^ induced membrane hyperpolarization and subsequent capillary relaxation. Beyond dynamic regulation of vascular tone, pericytes are essential for maintaining iBRB/BBB integrity through stabilization of tight junctions, controlling endothelial proliferation, and modulating angiogenic signaling [65,66]. These functions rely on intercellular communication between endothelial cells and pericytes, mediated by multiple pathways, including transforming growth factor-β (TGF-β), angiopoietins, platelet-derived growth factor-B (PDGF-B), sphingosine-1-phosphate (S1P), and Notch signaling. Moreover, pericytes exhibit multipotent differentiation capacity, with the ability to generate diverse cell types, positioning them as a key cellular target for tissue repair and regenerative therapies [62,67]. Conversely, the loss or dysfunction of pericytes, as seen in diabetic retinopathy, disrupts these signaling networks and leads to impaired neurovascular coupling, capillary nonperfusion, and iBRB breakdown, underscoring their critical role in fine-tuning retinal blood flow and maintaining vascular stability [62,68].

In summary, these signaling pathways link neuronal activity to vascular responses, ensuring precise neurovascular coupling and maintaining retinal homeostasis. While the retina and brain share core mechanisms such as NO-mediated vasodilation, ATP- and ion-dependent modulation, VEGF signaling, and pericyte regulation, the retina exhibits distinct adaptations: ATP/adenosine signaling is particularly prominent in the deep capillary plexus, NO signaling predominates in the superficial plexus, ionic (K^+^ and Ca^2+^) dynamics are tightly spatially restricted, VEGF primarily supports retinal vascular maintenance rather than angiogenesis under normal conditions, and pericytes display specialized roles in regulating capillary flow across stratified retinal layers (Table 1). When disrupted, they lead to mismatches between metabolic demand and blood supply, resulting in hypoxia, oxidative stress, and barrier breakdown that contribute to retinal diseases such as diabetic retinopathy, glaucoma, and age-related macular degeneration (AMD) and other neuro-dysfunctions.

## 4. Neurovascular Coupling Dysfunction in Disease

Over the past decades, extensive research has explored retinal neurovascular coupling dysfunction in retinal neurodegenerative diseases such as diabetic retinopathy, AMD, and glaucoma. The classical mechanisms underlying neurovascular coupling deficits in these conditions have been comprehensively reviewed in the previous literatures [60,72,73,74]. Building on this foundation, recent investigations have focused on neurovascular coupling impairments in major brain disorders, including Alzheimer’s disease, Parkinson’s disease, Huntington’s disease, and stroke. Given the structural and functional parallels between the retina and brain, noninvasive imaging of retinal neurovascular coupling provides a unique window into early cerebrovascular and neuronal dysfunction. Advanced modalities such as fOCT, OCT and OCTA enable high-resolution, label-free assessment of activity-dependent vascular responses and microvascular alterations in vivo. By detecting subtle changes in retinal perfusion and neural activity, these techniques hold great promise as non-invasive, early diagnostic and monitoring tools for neurovascular deficits associated with brain diseases.

### 4.1. Retinal Structure and Neurovascular Alterations in Alzheimer’s Disease

Although Alzheimer’s disease has traditionally been regarded as a brain disorder, growing evidence indicates that it also manifests in the eye, with detectable biomarkers in the retina, vitreous, cornea, and other ocular structures. One key pathological feature is impaired neurovascular coupling, well documented in the brain and also observed in the retina, reflecting their shared neurovascular pathophysiology [8,75,76]. Functional imaging and electrophysiological studies demonstrate reduced retinal blood flow responses to visual stimulation, indicating deficits in activity-dependent vascular changes in Alzheimer patients. These functional impairments are accompanied by structural and molecular changes within the retinal neurovascular unit, including pericyte loss or dysfunction, disruption of endothelial tight junctions, decreased expression of vasoactive mediators such as NOS, and altered gliotic responses of Müller cells and astrocytes [77,78]. Retinal vascular imaging in Alzheimer’s disease patients and animal models has revealed vessel narrowing, decreased vessel density, and altered capillary hemodynamics. fOCT and angiography (OCTA) and dynamic vessel analysis (DVA) further show diminished flicker light–induced vasodilation [79]. These findings support the use of multimodal retinal assessments as noninvasive approaches to detect neurovascular pathology in Alzheimer’s disease. In recent years, increasing attention has been given to tauopathy and amyloidosis within the retinal neurovascular unit, highlighting the retina as a potential window into early neurodegenerative processes. For example, tau accumulation in the retina promotes early neuronal dysfunction and precedes brain pathology in a mouse model of Alzheimer’s disease [80,81], demonstrating that retinal tau aggregation leads to synaptic and functional impairments before overt cortical pathology. This suggests that retinal tauopathy may serve as an early biomarker of central tau-related neurodegeneration. Similarly, amyloid-β (Aβ) deposition has been detected in the inner retinal layers and vascular walls of both Alzheimer’s disease patients and transgenic mouse models [82,83,84], implicating retinal amyloidosis in vascular dysregulation and neuronal loss. Studies have further shown that Aβ accumulation can disrupt the integrity of the iBRB and impair neurovascular coupling [76,85,86], which leads to altered retinal perfusion and neuronal dysfunction. Together, these findings indicate that tau and Aβ pathologies converge within the retinal neurovascular unit, contributing to early vascular and synaptic deficits that parallel those observed in the brain.

Beyond vascular alterations, retinal changes include microgliosis, Müller cell degeneration, thinning of the retinal nerve fiber layer, and ganglion cell loss, all of which have been proposed as potential biomarkers for early disease detection [87,88]. Building on these observations, OCT has gained attention as a promising tool for both early diagnosis and longitudinal monitoring of Alzheimer’s disease. Expanding on this potential, researchers are increasingly exploring retinal imaging as a noninvasive window into Alzheimer’s and dementia pathology. By leveraging high-resolution techniques such as OCT, OCTA, transmission electron microscopy (TEM), and ultra-spectral imaging, it is possible to detect subtle retinal changes—thinning of the nerve fiber layer, microvascular rarefaction, altered blood flow, and Aβ accumulation—that mirror in brain pathology [89,90]. These biomarkers may allow early identification of individuals at risk for Alzheimer’s, enabling timely interventions from lifestyle modifications to pharmacologic treatments that could delay onset, slow progression, and improve long-term outcomes.

Challenges and future directions in this research area have been increasingly recognized. While retinal imaging holds significant promise for detecting Alzheimer’s disease, further studies are needed to validate these biomarkers across larger cohorts and at different stages of disease progression. Therefore, standardizing imaging protocols, analytical algorithms, and diagnostic thresholds will be essential to ensure reproducibility and comparability across studies [91,92]. Moreover, understanding the exact cellular and molecular mechanisms underlying retinal manifestations of Alzheimer’s pathology, such as Aβ deposition, tau aggregation, microvascular dysfunction, and neuroinflammation, will be critical for identifying reliable therapeutic targets. Integrating retinal imaging with other biomarkers, including cerebrospinal fluid analysis, plasma assays, and neuroimaging, may also improve early detection and risk stratification [93,94]. Ultimately, translating these advances into accessible and cost-effective screening tools will require interdisciplinary collaboration among neuroscientists, ophthalmologists, engineers, and clinicians.

### 4.2. Using Biomarkers to Detect Early Signs of Parkinson’s Disease and Dementia

In Parkinson’s disease, hallmark structural and molecular changes include the loss of dopaminergic neurons and the accumulation of misfolded α-synuclein (α-syn) Lewy bodies in the substantia nigra pars compacta, both of which have also been reported in the retina [9,95]. Retinal neurovascular coupling dysfunction can be detected before significant structural changes appear, making it a potential early marker for Parkinson’s disease [96,97]. Indeed, reductions in superficial and deep capillary plexus densities have been observed in Parkinson’s disease patients, correlating with disease severity and retinal ganglion cell loss, consequently, leading to impair neurovascular coupling and attenuate visual stimulation-evoked blood flow [98,99]. Specific retinal alterations include the accumulation of misfolded α-syn aggregates in neurons and glia [100]. Mechanistically, α-syn aggregation may disrupt neurovascular signaling by impairing the release of NO and other vasoactive metabolites, thereby reducing vascular responsiveness. Additional contributors include pericyte loss, endothelial dysfunction, altered glial calcium signaling, and compromised neuronal metabolism, which together impair energy-dependent vascular regulation and neurovascular coupling [98,100]. Furthermore, a reduction in dopaminergic amacrine cells, which play critical roles in retinal signal processing and contrast sensitivity, has also been reported. Such alterations may contribute to impair visual function, including deficits in motion perception and contrast detection, which are commonly reported in Parkinson’s disease patients [101]. Retinal structural alterations, particularly the loss of amacrine and ganglion cells and thinning of the inner plexiform layer and peripapillary retinal nerve fiber layer, correlate with dopaminergic neuron loss in the substantia nigra, highlighting shared neurodegenerative mechanisms between the retina and brain [102]. This thinning can be quantitatively measured using OCT, a non-invasive, high-resolution imaging technique capable of detecting early, subclinical retinal changes, making it a promising biomarker for Parkinson’s disease progression and severity [103]. Importantly, these structural alterations often parallel functional deficits detected by electrophysiological methods such as electroretinography (ERG) or psychophysical tests assessing visual acuity, contrast sensitivity, and color discrimination [101]. In Parkinson’s disease, pattern ERG (PERG) recordings frequently reveal reduced amplitudes of P50- and N95-waves, reflecting impaired macular pathway function and ganglion cell activity, while full-field ERG may show altered oscillatory potentials linked to dopaminergic amacrine cell dysfunction [104]. Notably, some studies have reported partial recovery of ERG responses following dopaminergic therapy, suggesting that these measures may also serve as functional indicators of treatment efficacy [105]. Furthermore, since the loss of dopaminergic signaling in the retina can disrupt neurovascular coupling, dopamine plays a modulatory role in regulating retinal blood flow in response to visual stimuli [106]. This links Parkinson’s disease-associated neuronal changes to vascular dysregulation.

The retina offers a unique and accessible site to detect neurodegenerative changes that may precede motor symptoms in Parkinson’s disease. Emerging evidence suggests that retinal alterations, including structural thinning of the ganglion cell and inner plexiform layers, loss of dopaminergic amacrine cells, and accumulation of α-syn, may precede the clinical diagnosis [107,108]. While OCT-based structural changes are relatively well documented, evidence for dopaminergic cell loss and α-syn deposition in the prodromal stage remains preliminary and requires further longitudinal validation. Nevertheless, the early changes in the retina are being actively investigated as biomarkers for Parkinson’s disease, with the potential to enable earlier intervention and improved disease management [9]. Non-invasive imaging modalities such as OCT and OCTA provide rapid, cost-effective structural assessments, while complementary approaches, including functional imaging and electrophysiological tests (e.g., PERG, flicker ERG), can detect subtle deficits in visual processing and neurovascular coupling [97,109,110]. Future studies incorporating artificial intelligence (AI)-driven analysis and large OCT cohorts across different disease stages have the potential to provide a more comprehensive view of the disease progression. Moving forward, progress in this area will depend on standardizing imaging protocols, integrating multimodal structural and functional readouts, and validating their predictive value in longitudinal cohorts. Such advances could establish retinal imaging as a practical and scalable tool for early Parkinson’s disease detection and monitoring.

### 4.3. Detecting Huntington’s Disease Through the Retina

Huntington’s disease is classically defined as a neurodegenerative disorder of the striatum and cerebral cortex, caused by an expanded CAG repeat in the huntingtin gene (HTT) that encodes a polyglutamine (polyQ) tract in the huntingtin protein. Consequently, the mutant huntingtin (mHTT) with an extended polyQ sequence forms aggregates in cortical and striatal neurons, leading to cellular damage and death [111]. Increasing evidence indicates that the retina is also affected, providing a potential window into disease mechanisms and progression [10,112,113]. Both human studies and the transgenic Huntington’s disease mouse models (e.g., R6/2, YAC128, and zQ175) have demonstrated retinal structural and functional alterations, reinforcing the retina as a sensitive site of Huntington’s disease-associated neurodegeneration. These changes include thinning of the inner retinal layers, disrupted synaptic organization, and progressive loss of ganglion and photoreceptor cells. Specifically, photoreceptor involvement is evidenced by thinning of the outer nuclear layer and loss of nuclei in the zQ175 mice, as well as degeneration observed in the R6 models. Correspondingly, several OCT studies in humans report thinning of the inner retinal ganglion cell complex, mirroring the cellular alterations seen in mouse models [114,115]. Furthermore, retinal changes in the R6/1 mice emerge earlier in the outer retina than in the inner retina, beginning with cone opsin loss and progressing to widespread structural and functional deficits by 6 months [116]. Electrophysiological recordings, such as ERG, demonstrate deficits in both photopic and scotopic responses, suggesting dysfunction across multiple retinal cell types. The studies also localize this damage to the outer plexiform layer and link it to loss of synaptic connectivity between the outer nuclear layer and inner nuclear layer, consistent with the observed ERG *b*-wave abnormalities. However, since these alterations do not parallel the temporal spread of mutant huntingtin aggregates, the R6/1 retina may be more useful for investigating Huntington’s disease mechanisms and therapeutic strategies rather than serving as an early diagnostic marker [117,118]. Additionally, molecular studies have detected mutant huntingtin aggregates in retinal neurons and glia, accompanied by altered expression of neurotransmission-related genes, inflammatory markers, and components of the neurovascular unit [118].

Vascular abnormalities have emerged as a significant feature of Huntington’s disease, extending beyond neuronal degeneration to include microvascular compromise in the retina. OCTA studies in humans have revealed decreased capillary density, choroidal thinning, and changes in foveal perfusion in patients with Huntington’s disease, highlighting the retina’s sensitivity to vascular pathology in both the central nervous system and systemic circulation [119,120]. Complementary evidence from Huntington’s disease mouse models, including R6/1 and R6/2 strains, demonstrates early iBRB disruption, retinal gliosis, and progressive retinal structural and functional changes, suggesting that barrier integrity and microvascular regulation are impaired alongside neuronal degeneration [116,118]. However, some reports in human patients have been more controversial, showing only minimal abnormalities in the temporal retinal nerve fiber layer and no other detectable structural or functional retinal changes. This discrepancy suggests that current OCT and color vision tests may not yet serve as reliable biomarkers for Huntington’s disease [121].

Future studies should integrate fOCT, OCTA, and electrophysiology with AI-based analysis to detect early retinal changes in the disease. Large-scale longitudinal imaging in experimental and clinical settings should be prioritized to define retinal changes as reliable biomarkers for early disease detection and monitoring. Mechanistic studies in animal models exploring cellular drivers such as gliosis, vascular dysfunction, and neuronal loss will be critical for linking these imaging findings to underlying pathology.

### 4.4. Retinal Structure Changes in Stroke

Stroke induces both functional and structural alterations in the retina that mirror central neurovascular pathology [122]. In particular, retinal neurovascular coupling is disrupted, as shown by attenuated vessel dilation and delayed hemodynamic responses to visual or flicker stimulation, which reflect impaired communication between neurons, glia, and vascular cells. These abnormalities have been observed in stroke patients. To explore these mechanisms, several well-established animal models of stroke have been employed to examine how cerebrovascular injury impacts the retina, thereby providing a unique window into neurovascular dysfunction [123,124,125]. Among them, the middle cerebral artery occlusion (MCAO) model is the most widely used; it induces focal ischemia and leads to thinning of inner retinal layers, retinal ganglion cell death, and impaired ERG responses, including delayed or diminished *a*- and *b*-wave amplitudes. Notably, the severity of these ERG deficits correlates with infarct size, behavioral impairments, and ganglion cell loss, mirroring the extent of cortical damage [126]. In addition, MCAO triggers inflammatory responses in the retina, characterized by elevated cytokine levels and microglial activation following reperfusion [124]. The bilateral common carotid artery occlusion (BCCAO) model, which produces chronic cerebral hypoperfusion, results in progressive inner retinal degeneration, vascular remodeling, and glial activation, closely resembling features of small vessel disease and vascular cognitive impairment [127,128]. Similarly, unilateral common carotid artery occlusion (UCCAO) in mice induces retinal hypoxia, marked by HIF-1α stabilization, gliosis, and ERG deficits that emerge within days of occlusion [129]. Finally, photothrombotic stroke models, which allow spatially controlled vascular occlusion, have provided additional insight by revealing localized disruptions in retinal blood flow and neurovascular coupling [125,130].

To better understand how stroke-induced cerebrovascular events affect the retina, researchers have employed a wide range of imaging and experimental techniques to examine structural alterations, providing insights into the consequences of cerebral ischemia on retinal tissue [11,122]. Among these, OCT has been widely used to provide precise measurement of retinal layer thickness, particularly the retinal nerve fiber layer, ganglion cell complex, and inner retina, which reflect neuronal loss and often parallel cortical degeneration [109]. Building on this structural information, OCTA enables visualization of the retinal microvasculature without dye injection, revealing reduced vessel density, perfusion deficits, and remodeling of the superficial and deep vascular plexuses after ischemic events [131]. Fundus photography (FP) and fluorescein angiography (FA) continue to play a role in documenting gross retinal vascular abnormalities, such as hemorrhages or ischemic regions, though they provide less quantitative data than OCT-based approaches [132]. Complementing these imaging modalities, retinal vessel analysis (RVA) offers dynamic assessment of vascular reactivity to flicker light stimulation, highlighting impaired neurovascular coupling as a hallmark of stroke-induced dysfunction [14]. Dynamic RVA can detect reduced vessel diameter and blunted neurovascular responses in the acute phase, some of which partially recover over time, and these observations hold promise as potential biomarkers for early detection of stroke-related brain injury. Additionally, in experimental animal models, histological and immunohistochemical techniques provide cellular-level resolution, demonstrating neuronal degeneration, synaptic alterations, microglial activation, and vascular pathology in the retina post-stroke [124,133,134,135]. Complementary to these, ERG, while primarily functional, also supports structural interpretations by correlating inner retinal damage with reduced *a*- and *b*-wave amplitudes. Together, these methodologies, spanning in vivo imaging, vascular reactivity testing, and postmortem tissue analysis, form a robust toolkit for linking retinal structural changes to stroke pathophysiology, with growing potential for translation into clinical biomarkers.

The retina provides a non-invasive, accessible window to monitor stroke-related neurovascular and structural changes. Advances in OCT, OCTA, and ERG enable detailed assessment of retinal microvascular alterations and neuronal degeneration, supporting early detection and monitoring of stroke progression. Future research should prioritize standardizing retinal imaging protocols in stroke patients and evaluating the potential of retinal biomarkers to predict outcomes and guide therapeutic interventions.

In summary, studying Alzheimer’s disease, Parkinson’s disease, Huntington’s disease, and stroke through the retina offers a powerful window into neurovascular and neurodegenerative mechanisms shared with the brain. The retina, as an accessible extension of the central nervous system, exhibits early and measurable alterations in neuronal, glial, and vascular function that are parallel to central pathology. Retinal imaging and electrophysiological analyses reveal that Alzheimer’s disease is characterized by Aβ accumulation, vascular attenuation, and impaired neurovascular coupling; Parkinson’s disease shows dopaminergic dysfunction, mitochondrial stress, and microvascular rarefaction; Huntington’s disease demonstrates early synaptic and vascular deficits; and stroke leads to ischemic damage and blood-retina barrier disruption. Comparative analysis highlights both shared neurovascular unit dysfunction and disease-specific signatures in retinal neurons, glia, and microvessels, underscoring the retina’s potential as a noninvasive biomarker for CNS disease progression (Table 2).

## 5. Conclusions

The retina, as an extension of the central nervous system, offers a unique, accessible window into neurovascular health and disease. Retinal neurovascular coupling represents a highly coordinated interplay between neurons, glia, and vascular cells, ensuring that local blood flow dynamically matches metabolic demand [136,137]. Mechanistically, this process involves activity-dependent signaling pathways, including NO, ATP, arachidonic acid metabolites, VEGF and ions-mediated vascular responses, all of which are tightly regulated through neuron–glia–vascular interactions [136,138]. Disruption of these pathways is increasingly recognized in neurodegenerative and vascular disorders, with retinal neurovascular dysfunction reflecting parallel alterations in the brain [139]. Owing to its accessibility and structural homology with the brain, the retina offers a unique and non-invasive platform to monitor neurovascular health and identify biomarkers for early detection, disease progression, and therapeutic evaluation.

While fOCT, OCT, and OCTA provide powerful means to assess retinal microvascular structure and perfusion, these modalities primarily capture vascular parameters and do not directly measure neuronal or glial activity. Consequently, they yield only indirect information about neurovascular coupling, limiting mechanistic insight into how neural and glial signals regulate vascular responses. This is particularly significant because impaired neuron–vascular and glia–vascular communication occurs early in diseases such as Alzheimer’s, Parkinson’s, and stroke. Complementary approaches, including calcium or voltage imaging for neuronal activity and fluorescence-based or two-photon microscopy for astrocytic signaling, are therefore essential in basic and preclinical studies. Integrating these methods with fOCT and OCTA could provide a more comprehensive framework for understanding neurovascular coupling and its disruption in disease. Therefore, multidisciplinary approaches that integrate advanced imaging, electrophysiology, and molecular profiling will be vital to clarify neurovascular interaction mechanisms and bridge retinal findings to brain pathology.

Advancing our understanding of retinal neurovascular coupling holds significant promise for both basic neuroscience and clinical translation. Future studies should aim to dissect the molecular and cellular pathways that mediate neurovascular coupling, including neuron-to-glia and glia-to-vessel signaling, as well as the contributions of pericytes and endothelial heterogeneity [138,139]. The integration of high-resolution functional imaging, adaptive optics, and electrophysiological techniques will allow more precise mapping of activity-dependent vascular responses across retinal layers [140]. Clinically, longitudinal studies leveraging retinal imaging may provide sensitive biomarkers for early detection and progression monitoring in neurodegenerative and cerebrovascular diseases, complementing brain imaging and fluid-based diagnostics. Furthermore, exploring pharmacological or genetic modulation of retinal neurovascular coupling may reveal novel therapeutic avenues to protect neurovascular integrity, not only in the retina but across the CNS [140,141]. Ultimately, combining mechanistic insights with translational applications will establish the retina as a robust model for probing neurovascular health and dysfunction in human brain disease.

## Figures and Tables

**Table 1:** Summarizing shared and retina-distinct signaling pathways in neurovascular coupling for NO, ATP, ions, VEGF, and pericytes.

Signaling Pathway	Shared Features (Retina and Brain)	Retina-Specific Features/Distinctions
Nitric Oxide (NO)	Produced by nNOS/iNOS/eNOS in neurons, glial and endothelial cells, respectively; induces vasodilation and increases local blood flow	Layer-specific modulation across superficial, intermediate, and deep capillary plexuses; rapid responses to visual stimulation; higher integration with retinal glia (Müller cells)
ATP/ Purinergic Signaling	Released from neurons and glia; acts on A_1_/A_2_ and P_2X_/P_2Y_ receptors on pericytes and endothelial cells to modulate vascular tone	Stratified effect: deep plexus more sensitive to ATP/adenosine modulation; tightly coordinated with glial Ca^2+^ signaling; spatially precise regulation of blood flow to match synaptic activity
Ionic Regulation (K^+^, Ca^2+^)	Extracellular K^+^ and intracellular Ca^2+^ regulate vascular tone in both brain and retina; involved in neurovascular signaling	Layer-specific ionic dynamics mediated by Müller glia; K^+^ buffering and Ca^2+^ waves directly influence pericytes across capillary plexuses; rapid adjustments to localized visual activity
VEGF Signaling	Supports endothelial survival and angiogenesis; involved in adaptive vascular remodeling	Retina-specific VEGF gradients across layers; finely tuned to inner retinal metabolic demands; dysregulation leads to pathological angiogenesis (e.g., diabetic retinopathy, AMD) [69,70]
Pericytes	Modulate capillary diameter and blood flow; interact with endothelial cells via NO, ATP, and growth factors	Highly stratified and closely associated with synaptic layers; exhibit layer-specific contractility; integrate signals from neurons and Müller cells; coordinate local vascular remodeling; the retina is particularly susceptible to pericyte loss–related vascular leakage [5,71]

**Table 2:** Comparative summary of retinal neurovascular dysfunction across AD, PD, HD, and stroke.

Disease	Key Retinal Neuronal Changes	Glial/Microglial Responses	Vascular Alterations	Distinct Retinal Features/Biomarkers
Alzheimer’s disease (AD)	Loss of RGCs; synaptic dysfunction; Aβ and tau deposition	Reactive gliosis (Müller and astrocytes); microglial activation	Loss of pericytes; vasoconstriction; reduced flow; iBRB disruption	Retinal thinning (OCT); Aβ plaques; impaired flicker-induced vasodilation
Parkinson’s disease (PD)	Dopaminergic amacrine cell loss; altered contrast sensitivity; α-syn aggregates	Elevated oxidative stress and glial reactivity	Microvascular rarefaction; reduced capillary density	ERG deficits; reduced retinal dopamine markers
Huntington’s disease (HD)	Progressive loss of photoreceptors and RGCs; early synaptic loss in the inner retina	Microglial activation; increased inflammatory glial signaling [116]	↓ Capillary density; vascular remodeling and leakage	Reduced *b*-wave amplitude; impaired NVU response; thinning of retina
Stroke/ Ischemic injury	Loss of nerve fibers; ischemic RGC death; inner retinal layer thinning	Astrocyte and Müller glia swelling; microglial recruitment	iBRB disruption; vessel occlusion; reduced autoregulation	ERG deficits; Inner retinal infarcts (OCT); NVU uncoupling in post-stroke recovery

## Data Availability

Not applicable.

## Abbreviations

The following abbreviations are used in this manuscript:

NVU	Neurovascular unit
RGCs	Retinal ganglion cells
iBRB	inner blood–retina barrier
oBRB	outer blood–retina barrier
BBB	Blood–brain barrier
OCT	Optical coherence tomography
OCTA	Optical coherence tomography
NO	Nitric oxide
NOS	Nitric oxide synthase
ATP	Adenosine triphosphate
cAMP	Adenosine monophosphate
PKA	Protein kinase A
PKC	Protein kinase C
MAPK	Mitogen-activated protein kinase
PLC	Phospholipase C
PGE_2_	prostaglandin E_2_
EETs	epoxyeicosatrienoic acids
